# Is Hepatocyte Necrosis a Good Marker of Donor Liver Viability During Machine Perfusion?

**DOI:** 10.1002/hep4.1816

**Published:** 2021-09-02

**Authors:** Desley A.H. Neil, Hynek Mergental, Angus Hann, Richard W Laing, Hermien Hartog, Darius F Mirza, M. Thamara P.R. Perera

**Affiliations:** ^1^ Department of Cellular Pathology University Hospitals Birmingham NHS Foundation Trust Birmingham United Kingdom; ^2^ Centre for Liver and Gastrointestinal Research Institute of Immunology and Immunotherapy University of Birmingham Birmingham United Kingdom; ^3^ Liver Unit Queen Elizabeth Hospital University Hospitals Birmingham NHS Foundation Trust Birmingham United Kingdom; ^4^ National Institute for Health Research, Birmingham Biomedical Research Centre University of Birmingham and UHBFT Birmingham United Kingdom

TO THE EDITOR:

We read with interest the paper by Kesseli et al.,^(^ ^1^ ^)^ which uses a primate nontransplant model to assess markers of nonviability of donation after circulatory death (DCD) livers during normothermic machine perfusion (NMP). They use histological assessment of hepatocyte necrosis as the basis of determining whether a liver is nonviable and conclude that lactate clearance during NMP is not able to differentiate between viable and nonviable livers.

We question this approach based on our clinical experience:

1. We have transplanted several donation after brain death (DBD) livers turned down for standard transplantation because of high donor aspartate aminotransferase levels, which underwent NMP and were assessed using viability criteria based on lactate clearance.^(^ ^2^ ^)^ Two of these cases are published to demonstrate not only that they do function following transplantation, but are suitable for high‐risk recipients.^(^ ^3^ ^)^ Over 50% and 25% hepatocyte necrosis was present in the liver transplanted into the stable and unstable recipient, respectively.
2. In the VITTAL clinical trial,^(^ ^4^ ^)^ 31 livers (14 DCDs and 17 DBDs), turned down by UK units for standard transplantation, were assessed by NMP using lactate clearance–based viability criteria. We found no significant difference in the amount of hepatocyte necrosis after 4 hours on NMP between the seven livers that failed viability criteria median (range) 0% (0%‐40%) compared with the 24 that passed viability criteria 1% (0%‐30%), with up to 30% hepatocyte necrosis present in livers transplanted successfully. However, within the DCD cohort, which had a median donor warm ischemia time of 21 (11‐46) minutes and static cold storage time of 7 hours (5.5‐10), the four livers failing viability criteria had significantly more hepatocyte necrosis 20% (5‐40) than the 10 DCDs that passed viability criteria and were transplanted 1% (0‐5); *P* < 0.023.

In conclusion, significant hepatocyte necrosis in DBD grafts can be successfully transplanted following NMP, indicating that hepatocyte necrosis *per se* is not a reliable surrogate for viability of a donor liver. Viability criteria based on lactate clearance can identify hepatocyte necrosis in DCD livers. This suggests that different mechanisms of injury and repair are involved in DBD and DCD grafts.
